# Supplementary material for: Determination of X-ray detection limit and applications in perovskite X-ray detectors
Source: Nat Commun. 2021 Sep 6;12:5258. doi: 10.1038/s41467-021-25648-7 (PMC8421435; doi:10.1038/s41467-021-25648-7)
Supplement: Supplementary file 1 — Supplementary information [file 41467_2021_25648_MOESM1_ESM.pdf]

Supplementary Information

Determination of X-ray Detection Limit and Applications in Perovskite X-ray Detectors

Lei Pan[1], Shreetu Shrestha[2], Neil Taylor[1], Wanyi Nie[2] and Lei R. Cao[1*]

[1]*Nuclear Engineering Program, Department of Mechanical and Aerospace Engineering, The Ohio State University, Columbus, Ohio, 43210, USA*

[2]*Center for Integrated Nanotechnology Materials Physics and Application Division, Los Alamos National Laboratory, Los Alamos, NM, 87545, USA*

*Correspondence to: cao.152@osu.edu

Supplementary Table 1. $MAPbI_3$ device dimensions

	#1 Au/Au	#2 Pb/Au	#3 Pb/Au	#4 Pb/Au
$MAPbI_3$ thickness (mm)	1.0	1.3	0.6	0.9
Electrode area (mm^2)	9.28	4.68	10.50	6.82
Electrode thickness (nm)	100	100	100	100

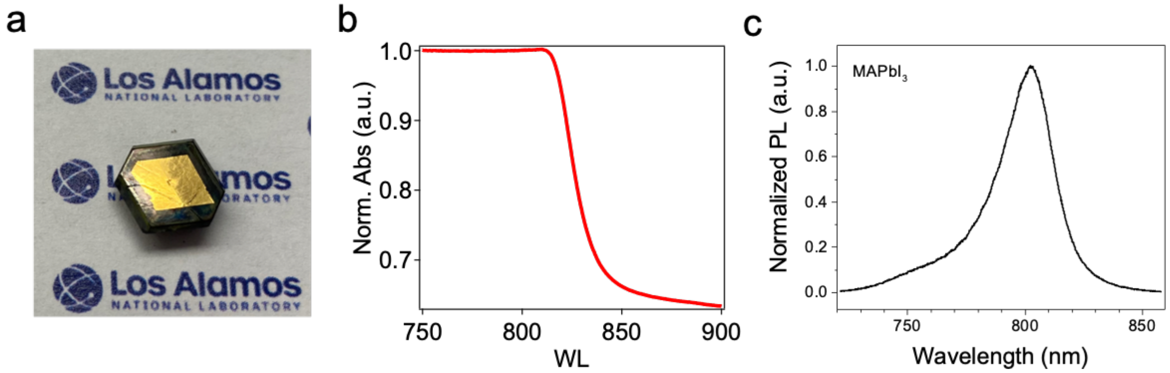

Supplementary Fig. 1. $MAPbI_3$ Single crystal characterizations. a. photo of a typical single crystal device. **b.** absorption spectrum and **c.** photoluminescence spectrum for $MAPbI_3$ single crystal.

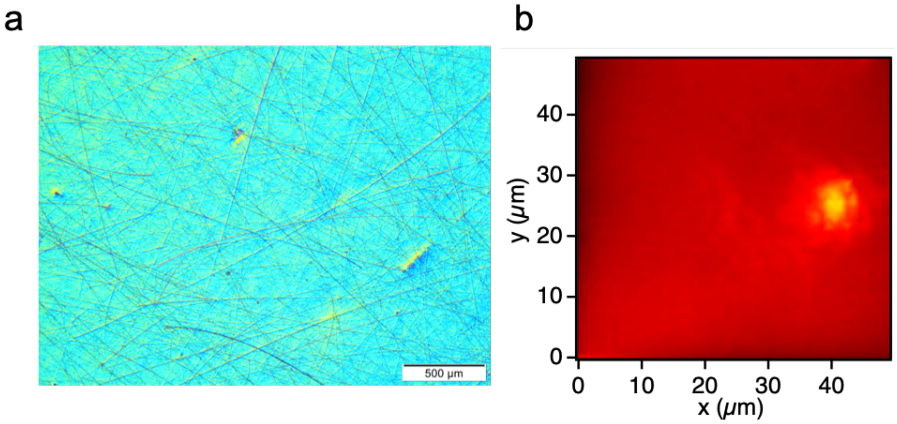

Supplementary Fig. 2. Surface characterizations for the $MAPbI_3$ single crystal. a. optical microscope image for $MAPbI_3$ single crystal's surface after polishing. **b.** photoluminescence height map for a typical crystal after polishing.

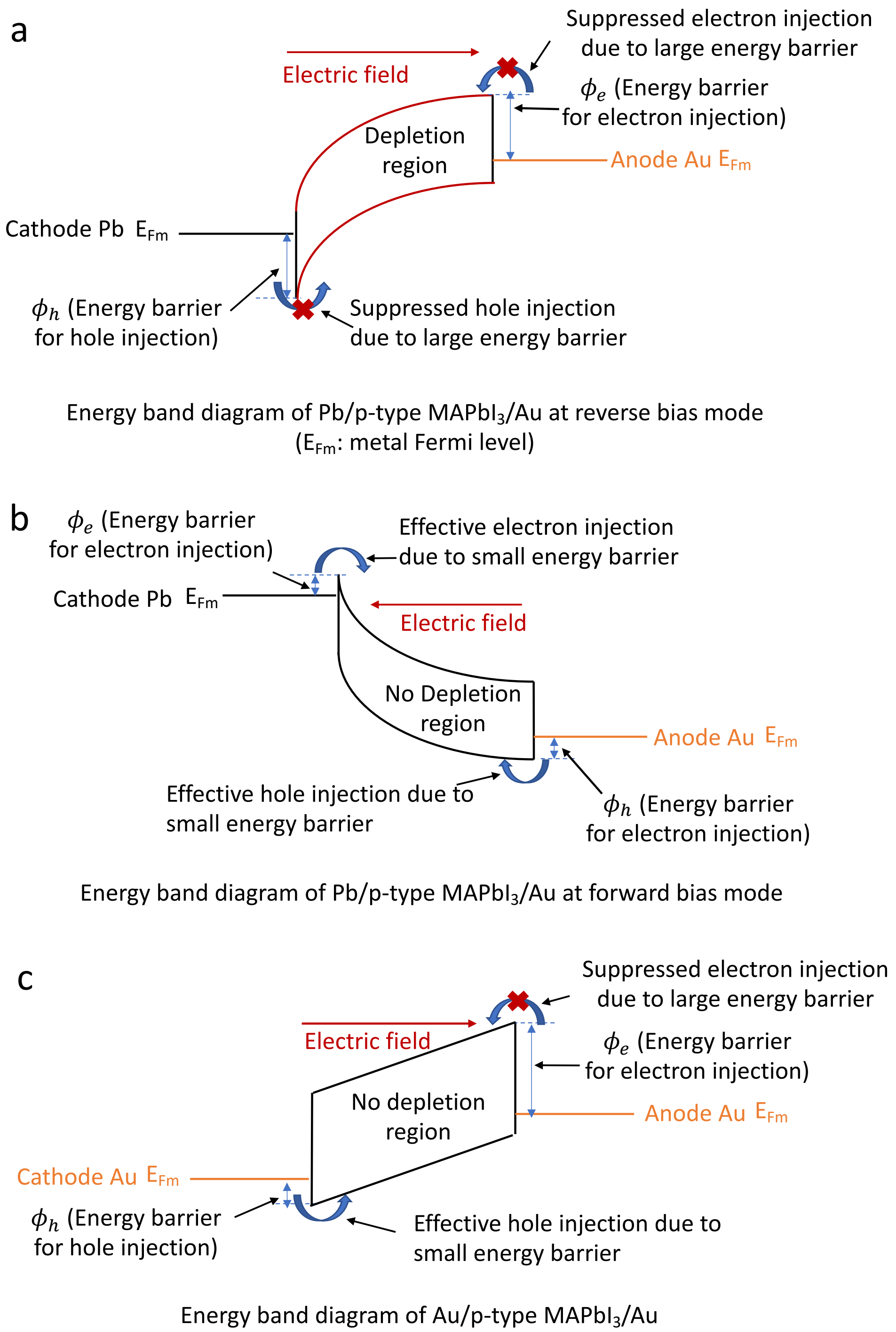

Supplementary Fig. 3. Energy band diagram of different device architectures for charge injection analysis. a. Pb/p-type $MAPbI_3$/Au at reverse bias mode. **b.** Pb/p-type $MAPbI_3$/Au at forward bias mode. **c.** Au/p-type $MAPbI_3$/Au

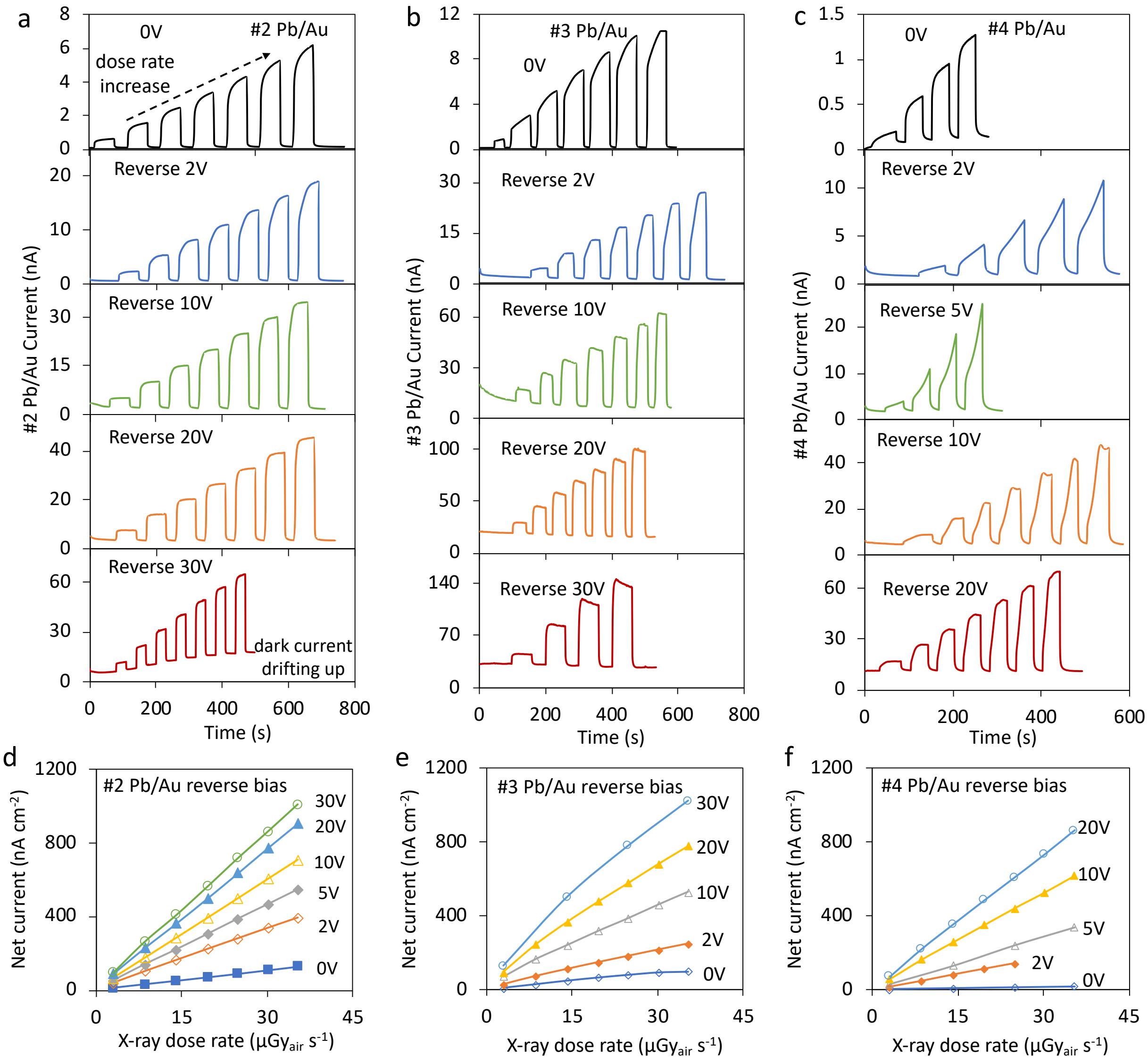

Supplementary Fig. 4. Sensitivity measurement of reversely biased Pb/Au devices. Current response (hole-dominantly induced signal) of **a.** #2 Pb/Au device, **b.** #3 Pb/Au device **c.** #4 Pb/Au device, to increased X-ray dose rate in air at different reverse bias voltage. Net current density as function of X-ray dose rate in air of **d.** #2 Pb/Au device, **e.** #3 Pb/Au device **f.** #4 Pb/Au device, at different reverse bias voltage.

Net current is calculated as the difference between the photocurrent under X-ray irradiation and the dark current. Sensitivity is obtained as the slope of linear fitting to net current density as function of X-ray dose rate in air.

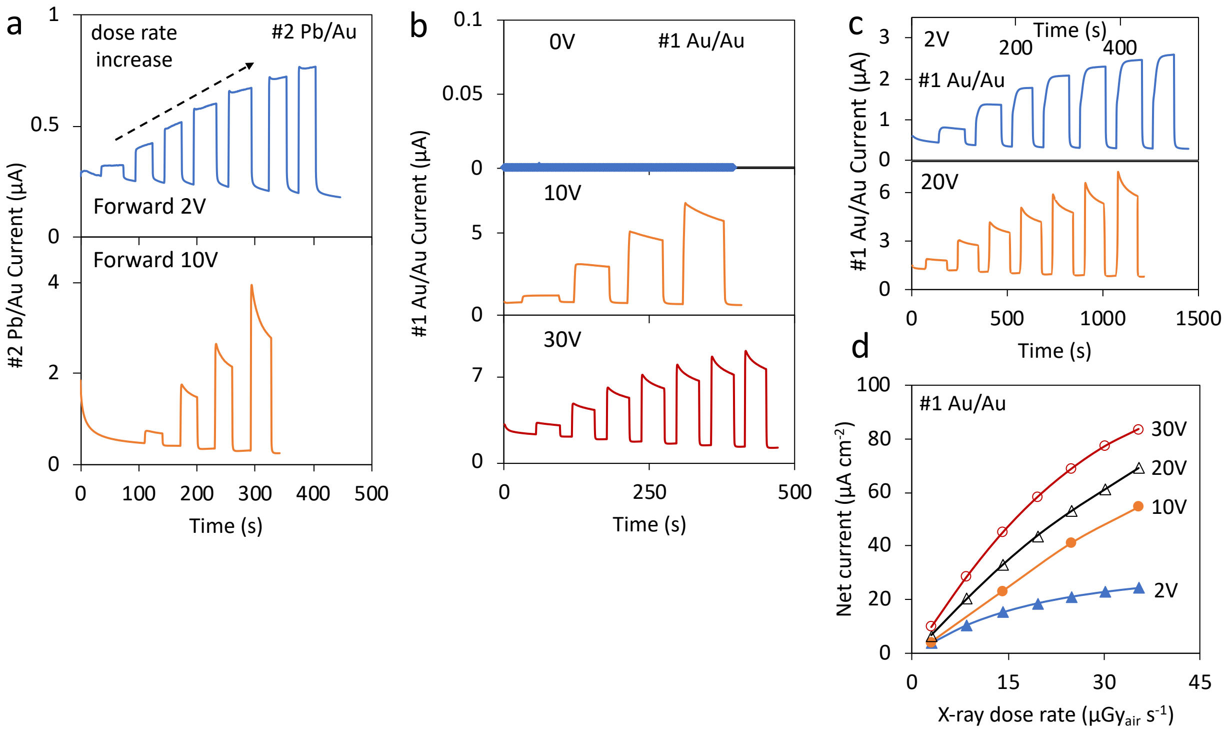

Supplementary Fig. 5. Sensitivity measurement of forward biased Pb/Au devices and Au/Au device. Current response (hole-dominantly induced signal) of **a.** forward biased #2 Pb/Au device, **b. c.** #1 Au/Au device, to increased X-ray dose rate in air at different voltage. **d.** Net current density as function of X-ray dose rate in air of #1 Au/Au device.

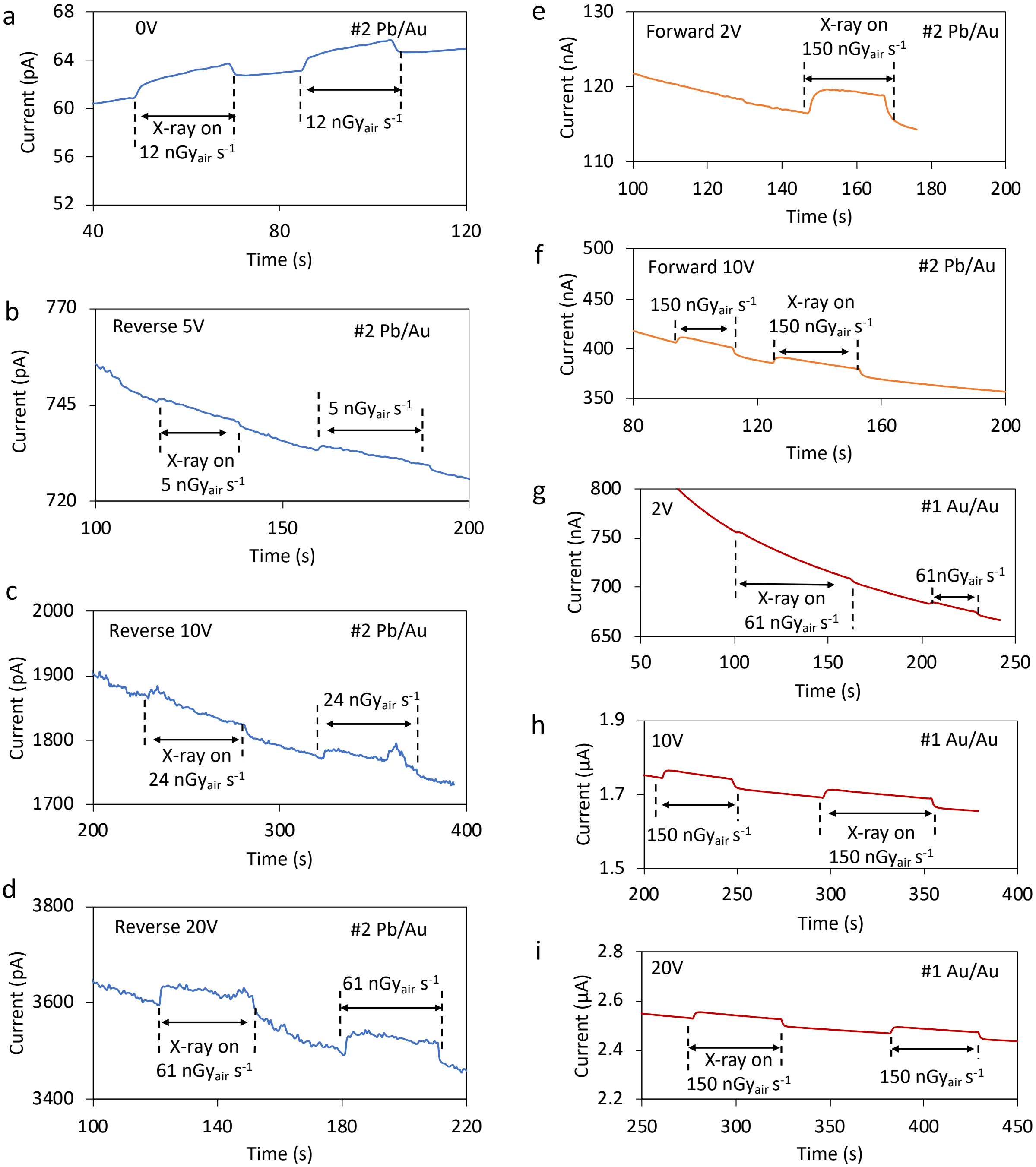

Supplementary Fig. 6. Measured detection limit of X-ray dose rate by X-ray photocurrent method. a. b. c. d. Current response of reversely biased #2 Pb/Au device at different reverse voltage. **e. f.** Current response of forward biased #2 Pb/Au device at different forward voltage. **g. h. i.** Current response of #1 Au/Au device at different voltage.

With our experiment setup, the X-ray dose rates available are 5 nGy$_{air}$ s^{-1}, 12 nGy$_{air}$ s^{-1}, 24 nGy$_{air}$ s^{-1}, 61 nGy$_{air}$ s^{-1}, 150 nGy$_{air}$ s^{-1}. The X-ray dose rates shown in the figures are the smallest detectable dose rates determined by the X-ray photocurrent method.

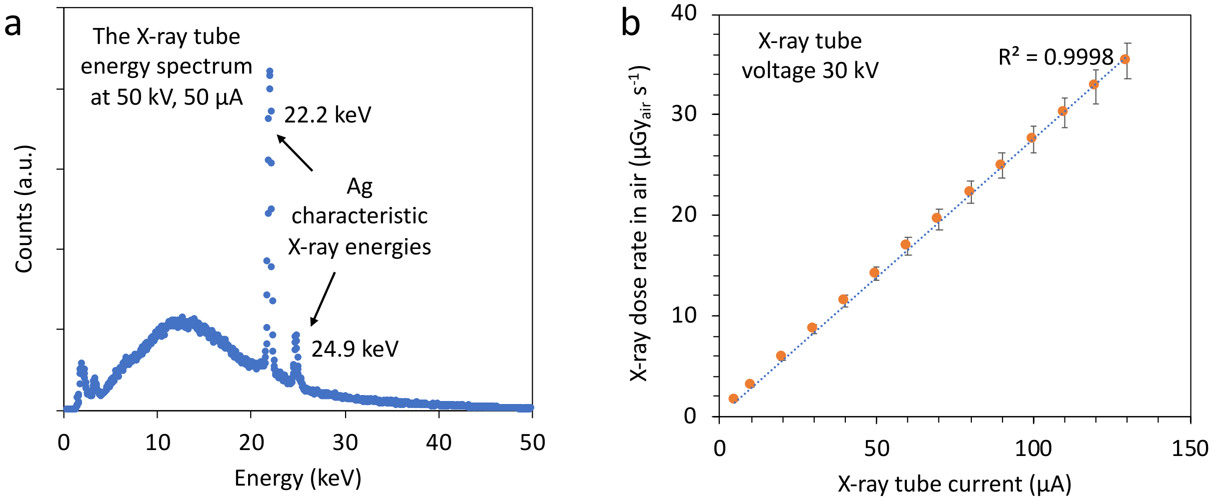

Supplementary Fig. 7. X-ray tube (with Ag target) energy spectrum and dose rate calibration
a. X-ray tube energy spectrum at tube voltage 50 kV and tube current of 50 µA, measured by a Si P-I-N detector. **b.** X-ray tube dose rate in air at tube voltage 30 kV and different tube current from 5 µA to 130 µA. A dosimeter (Fluke Biomedical RaySafe 452) was used for dose rate calibration. Error bars represent ±5% variation of the measured dose rate.

The X-ray tube has a most probable X-ray photon energy of 22.2 keV, corresponding to one of the Ag characteristic X-ray energies when the X-ray tube is operated at voltage higher than 22.2 kV.

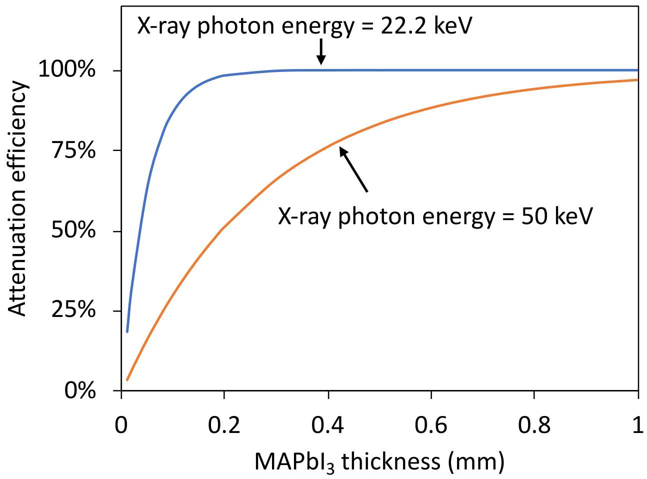

Supplementary Fig. 8. X-ray photon attenuation in $MAPbI_3$ signal crystal.

Attenuation efficiency = 1 - $e^{-(\frac{\mu}{\rho})\rho x}$, where $\frac{\mu}{\rho}$ is the mass attenuation coefficient of $MAPbI_3$, ρ is the density of $MAPbI_3$, and x is the $MAPbI_3$ single crystal thickness. The mass attenuation coefficient of $MAPbI_3$ can be calculated as $(\frac{\mu}{\rho})_{MAPbI3} = \sum_i w_i\,(\frac{\mu}{\rho})_i$ where w_i and $(\frac{\mu}{\rho})_i$ are the fraction by weight and the mass attenuation coefficient of the ith atomic constituent, respectively.

The attenuation efficiencies of $MAPbI_3$ to two X-ray photon energies are calculated. The 22.2 keV and 50 keV are the most probable energy and the maximum energy, respectively, of the X-ray tube used in this work (see **Supplementary Fig. 7**). It can be effectively treated that the charge carriers are generated at the surface of the $MAPbI_3$ single crystal due to the limited penetration depth of the X-ray photons, *i.e.*, less than 0.2 mm for 22.2 keV X-ray photons.

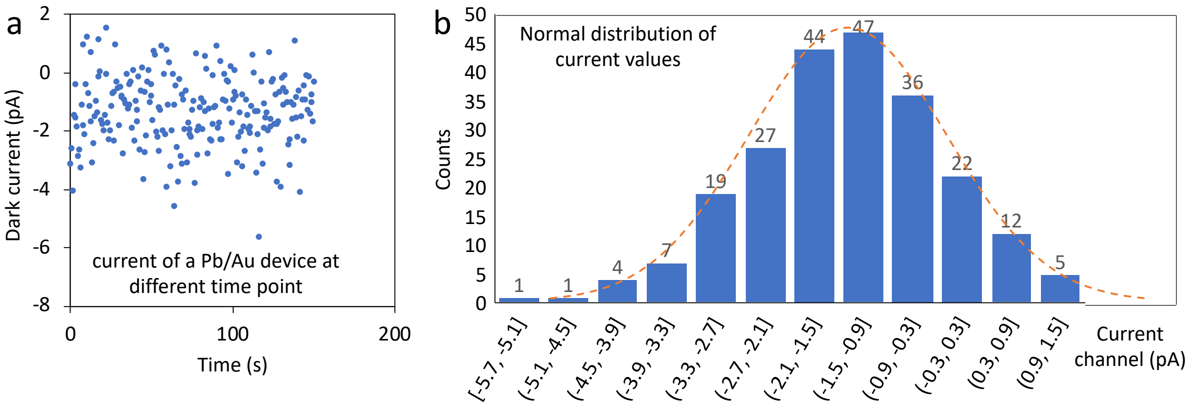

Supplementary Fig. 9. Statistical distribution of the current values. a. dark current of a Pb/Au device measured as a function of time. The measurement was performed at 0V to avoid the interference of dark current drift. **b.** Current value distribution fitting to Normal distribution, supporting that dark current follows Normal distribution.
